# ML218 HCl Is More Efficient Than Capsaicin in Inhibiting Bacterial Antigen-Induced Cal 27 Oral Cancer Cell Proliferation

**DOI:** 10.3390/ijms222212559

**Published:** 2021-11-22

**Authors:** Rajdeep Chakraborty, Honghua Hu, Charbel Darido, Karen Vickery, Shoba Ranganathan

**Affiliations:** 1 Faculty of Medicine Health and Human Sciences, Macquarie University, Sydney, NSW 2109, Australia; rajdeep.chakraborty@hdr.mq.edu.au (R.C.); karen.vickery@mq.edu.au (K.V.); 2 Faculty of Science and Engineering, Macquarie University, Sydney, NSW 2109, Australia; 3 Peter MacCallum Cancer Centre, Melbourne, VIC 3000, Australia; charbel.darido@petermac.org; 4 Sir Peter MacCallum Department of Oncology, The University of Melbourne, Parkville, VIC 3010, Australia

**Keywords:** lipopolysaccharide, lipoteichoic acid, oral cancer, ML218 HCl, capsaicin, proliferation, apoptosis

## Abstract

The bacterial antigen, lipopolysaccharide (LPS) and disruptions in calcium channels are independently known to influence oral cancer progression. Previously, we found that bacterial antigens, LPS and lipoteichoic acid (LTA) act as confounders during the action of capsaicin on Cal 27 oral cancer proliferation. As calcium channel drugs may affect oral cancer cell proliferation, we investigated the effect of ML218 HCl, a T-type voltage-gated calcium channel blocker, on the proliferation of Cal 27 oral cancer cells. We hypothesized that ML218 HCl could effectively reduce LPS-induced oral cancer cell proliferation. LPS and LTA antigens were added to Cal 27 oral cancer cells either prior to and/or concurrently with ML218 HCl treatment, and the efficacy of the treatment was evaluated by measuring Cal 27 proliferation, cell death and apoptosis. ML218 HCl inhibited oral cancer cell proliferation, increased apoptosis and cell death, but their efficacy was significantly reduced in the presence of bacterial antigens. ML218 HCl proved more effective than capsaicin in reducing bacterial antigen-induced Cal 27 oral cancer cell proliferation. Our results also suggest an interplay of proliferation factors during the bacterial antigens and calcium channel drug interaction in Cal 27. Bacterial antigen reduction of drug efficacy should be considered for developing newer pharmacological agents or testing the efficacy of the existing oral cancer chemotherapeutic agents. Finally, voltage gated calcium channel drugs should be considered for future oral cancer research.

## 1. Introduction

Cancer of the oral cavity is one of the most prevalent cancers worldwide, resulting in high morbidity and mortality rates [1].

We recently reported that the combination of bacterial antigens, lipoteichoic acid (LTA) and lipopolysaccharide (LPS), resulted in increased proliferation of the oral cancer cell Cal 27, but not SCC4, SCC9 and SCC25, compared to stimulation with LPS alone [2]. We then developed an in vitro assay to measure the efficacy of drug treatment on Cal 27 in the presence of bacterial antigens, and examined the effect of capsaicin, which is known to induce apoptosis in several cancers [3]. The bacterial antigens LPS and LTA decreased the anti-cancer efficacy of capsaicin, both for antigenic stimulation given prior to and concurrently with this drug [3].

Calcium channels are linked directly or indirectly to all the ‘hallmarks of cancer’ [4]. Perturbation of calcium signalling occurs due to the pronounced changes in (a) expression levels, (b) altered cellular localisation, (c) altered post-translational modifications, and (d) genetic mutations. Changes in the activity of calcium channels lead to alterations in the calcium flux across plasma membrane and intracellular organelles in cancer cells, relative to their normal counterparts. The influx of calcium across the plasma membrane triggers tumour proliferation, migration, differentiation, and apoptosis [4]. Calcium oscillations thus can result in both proliferation and apoptosis of cancer cells via the calcium endoplasmic reticulum–mitochondrial calcium transfer axis [5,6].

LPS treatment induces Ca^2+^ entry in cancer cells in a TLR4-dependent manner, resulting in a chronic elevation of basal intracellular calcium levels [7]. Previous studies have shown that *Porphyromonas gingivalis* LPS tend to increase the viability of oral cancer cells [8], while within the tumour microenvironment, macrophage expression of IL-6 and CD14 is increased as well as free radical secretion [7]. Bacterial infection has been shown to be associated with chronic inflammation, anti-apoptotic activity, and pro-proliferative activities [9]. In our previous research we used capsaicin since capsaicin (TRPV1 agonist) was reported to impede proliferation and cause apoptosis in oral cancer cells SCC25 and SCC4 [10].

Xiang et al. reported ML218 as a T-type voltage-gated calcium channel [Cav 3.2 and Cav 3.3] inhibitor [11]. This raised the question as to whether substituting ML218 HCl (the commercially available form of ML218) for capsaicin in our drug treatment protocol [3] would lower Cal 27 oral cancer viability.

We hypothesized that ML218 HCl could be more effective than capsaicin in reducing bacterial antigen (LPS + LTA)-induced oral cancer cell proliferation and, therefore, the anti-cancer effects of ML218 HCl would be affected less by the presence of bacterial antigens compared to capsaicin (which we previously investigated [3]). This study focuses on investigating (a) the effect of ML218 HCl on Cal 27 oral cancer cell proliferation in the presence and absence of oral bacterial antigens, and (b) the effect of ML218 HCl on Cal 27 oral cancer cell proliferation factors SOCS3, EGFR, STAT3, PIK3 and TNFα, and apoptotic pathways in the presence and absence of oral bacterial antigens. Throughout this report, we included capsaicin data (that was previously published) [3] in each figure for ease of comparison between ML218 HCl and capsaicin treatment groups.

## 2. Results

### 2.1. Titration of ML218 HCl

**Figure 1:**
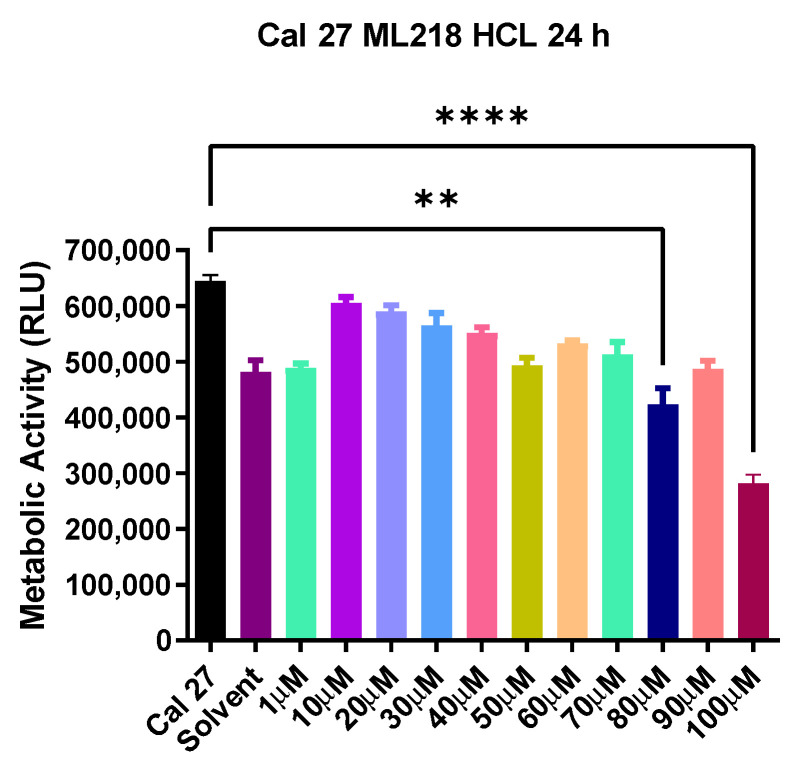
ML218 HCl drug titration. Metabolic activity of Cal 27 cells when treated with ML218 HCl for 24 h; Solvent is 0.1% DMSO used to solubilize the drug. Error bars represent standard error of the mean. ** *p* ≤ 0.01 and **** *p* ≤ 0.0001. RLU represents relative luminescence unit.

ML218 HCl cell viability was first determined (as detailed in Materials and Methods). The maximum amount of ML218 HCl tested against Cal 27 cells was 100 µM, as dosages above this level adversely affected normal cells, (results not shown) and therefore would not be clinically relevant. ML218 HCl at 100 µM produced maximum inhibition of Cal 27 oral cancer cells (*p* < 0.0001) (Figure 1). There was no significant difference observed in the cell metabolism of untreated Cal 27 and solvent treated Cal 27.

### 2.2. Effect of ML218 HCl Treatment on Normal Oral Cell OKF6 Metabolism and Viability

**Figure 2:**
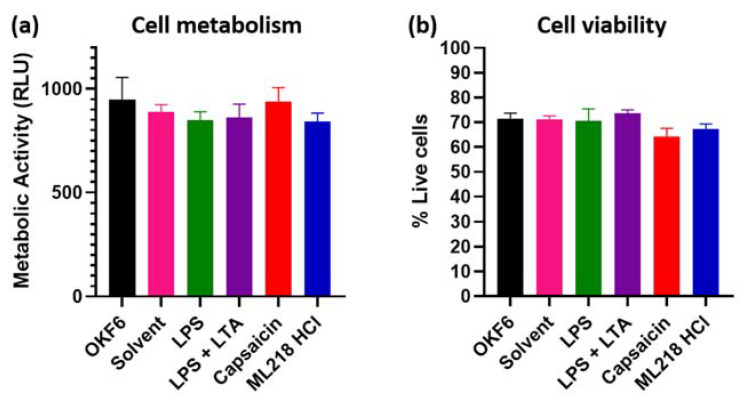
Bacterial antigens, ML218 HCl and capsaicin treatment on normal oral cell OKF6 metabolism and viability. OKF6 cells were plated at 5000 cells/well and incubated with drug solvent (0.1% DMSO), LPS (5 µg/mL), combined LPS (5 µg/mL) + LTA (5 µg/mL), capsaicin (150 µM) [3], and ML218 HCl (100 µM) for 24 h. (**a**) Cell metabolism quantified by the MT Glo assay. RLU = relative luminescence unit. (**b**) Cell viability estimated by the Trypan blue exclusion assay. *n* = six biological replicates. Error bars represent standard error of the mean.

We then checked the effect of ML218 HCl on normal oral cells, using the OKF6 cell line. There was no significant change in OKF6 metabolism and viability when treated with ML218 HCl, even at the maximum concentration determined 100 µM for Cal 27) (Figure 2). The results of ML218 HCl at 100 µM are similar to stimulation by bacterial antigens and by the maximum Cal 27 inhibitory dosage of capsaicin (150 µM [3]).

### 2.3. Effect of ML218 HCl Treatment on Oral Cancer Cell Metabolism and Viability in the Presence or Absence of Bacterial Antigens

**Figure 3:**
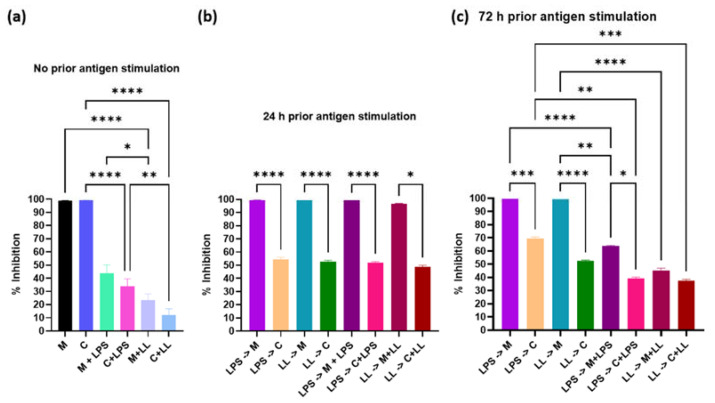
Effect of 100 µM ML218 HCl and 150 µM capsaicin [3] on Cal 27 cell metabolism. Cal 27 cells were either with (**a**) no antigen pre-stimulation, (**b**) 24 h or (**c**) 72 h bacterial antigens pre-stimulation prior to the addition of inhibitory drugs with and without additional antigen. M = ML218 HCL, C = capsaicin, LPS = lipopolysaccharide, LL = LPS + LTA (lipoteichoic acid). Letter before the arrow represents pre-stimulation, e.g., LPS -> M is LPS pre-stimulation and then treatment with ML218 HCL (M). *n* = nine biological replicates. Error bars represent standard error of the mean. * *p* ≤ 0.05, ** *p* ≤ 0.01, *** *p* ≤ 0.001, **** *p* ≤ 0.0001.

In the absence of antigenic stimulation, for 24 h following drug treatment, ML218 HCl reduced metabolism of Cal 27 cells by 100% (Figure 3a), similar to results obtained for capsaicin [3]. The addition of antigens along with ML218 HCl without prior antigenic stimulation significantly reduced metabolic inhibition (*p* ≤ 0.001) and this effect was significantly greater with the combined bacterial antigen treatment (LPS + LTA) than with LPS stimulation alone (*p* ≤ 0.001) (Figure 3a). This result was concordant with our earlier results for capsaicin [3].

When Cal 27 cells were provided antigenic stimulation prior to treatment, following ML218 HCl treatment, metabolism was inhibited by 100% (Figure 3b,c). In contrast, the addition of antigens prior to capsaicin treatment significantly reduced metabolic inhibition 30–50% (*p* ≤ 0.001) (Figure 3b,c). On the other hand, the addition of antigen concurrently with capsaicin treatment (values from [3]) significantly improved metabolism (*p* ≤ 0.001) (Figure 3b,c).

**Figure 4:**
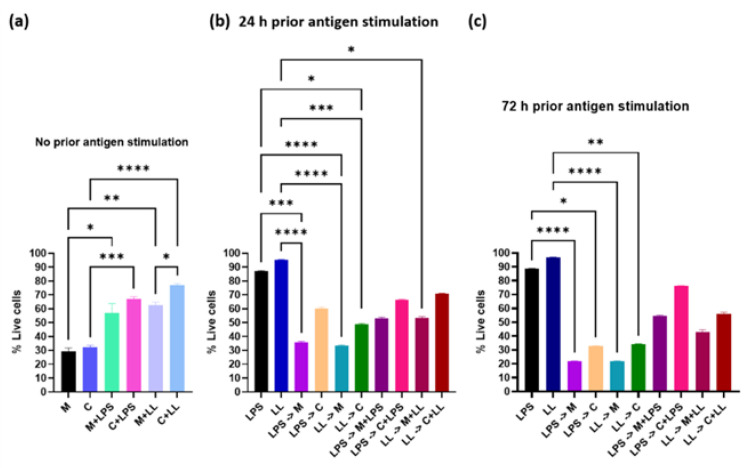
Effect of 100 µM ML218 HCl compared to 150 µM capsaicin [3] on Cal 27 cell viability. Cal 27 cells were cultured either with (**a**) no antigen pre-stimulation or (**b**) 24 h or (**c**) 72 h bacterial antigens pre-stimulation prior to the addition of inhibitory drugs in the presence or absence of additional antigen. M = ML218 HCL, C = capsaicin, LPS = lipopolysaccharide, LL = LPS + LTA (lipoteichoic acid). Letter before the arrow represents pre-stimulation, e.g., LPS -> M is LPS pre-stimulation and then treatment with ML218 HCL (M). n = nine biological replicates. Error bars represent standard error of the mean. * *p* ≤ 0.05, ** *p* ≤ 0.01, *** *p* < 0.001, **** *p* < 0.0001.

Cell viability results showed that in the absence of antigenic stimulation during the 24 h of drug treatment, ML218 HCl killed 70% of Cal 27 cells, compared to 68% for capsaicin [3] (Figure 4a). For ML218 HCl, providing antigenic stimulation prior to treatment but not during treatment gave similar results, with 70–80% of the cells killed (Figure 4b,c). However, when the cells were treated concurrently with bacterial antigen stimulation, the cell viability increased, with ML218 HCl still killing more cells (~40%) compared to capsaicin (~25%) (*p* ≤ 0.05) (Figure 4a). Cell viability was significantly higher in cultures with antigen added prior to and concurrently with treatment drugs when compared with cultures treated with each drug alone. This again suggests that the presence of antigens during treatment reduces the effectiveness of the drug. The percentage of cells killed was 20% higher among ML218 HCl treatment groups, compared to capsaicin [3] when antigens were added prior to and concurrently with the drugs (Figure 4b,c).

### 2.4. Modulation of ML218 HCl Induction of Apotosis by Bacterial Antigens

**Figure 5:**
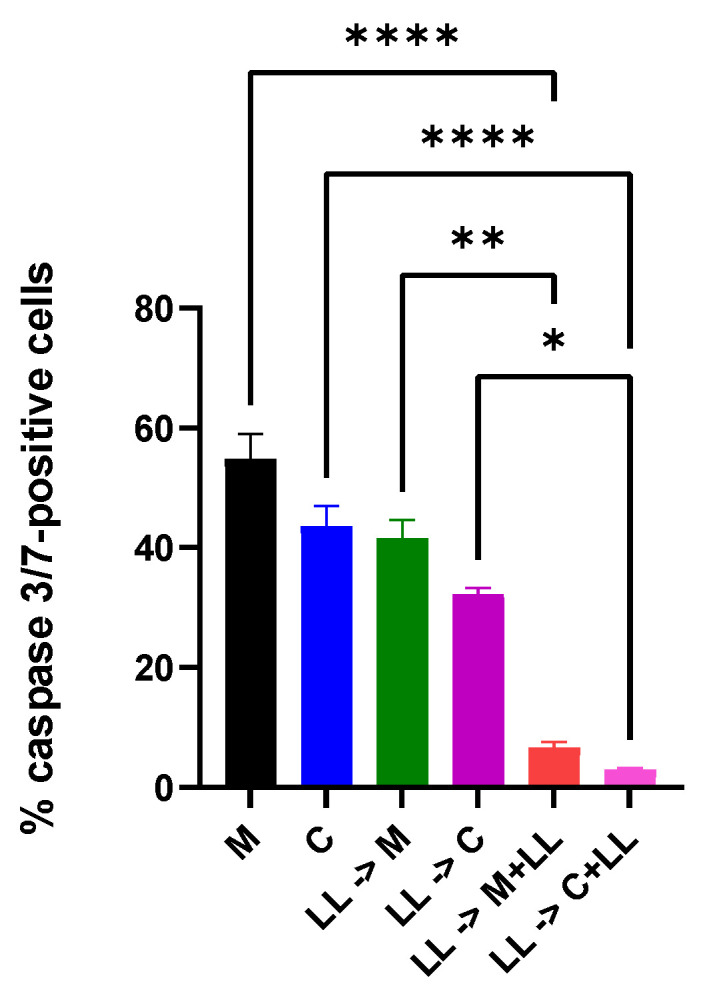
Percentage of Cal 27 apoptotic cells as determined by caspase 3/7-positivity. After Cal 27 cells were stimulated with LPS + LTA for 72 h, Cal 27 cells were treated with ML218 HCl and capsaicin [3] for 24 h in the absence or presence of oral bacterial antigens. LPS (5 µg/mL), LL = combined LPS (5 µg/mL) + LTA (5 µg/mL), M = ML218 HCl, C = capsaicin [3]. Letter before the arrow represents pre-stimulation, e.g., LPS -> M is LPS pre-stimulation and then treatment with ML218 HCl. * *p* ≤ 0.05, ** *p* ≤ 0.01, **** *p* ≤ 0.0001 are statistically significant. *n* = nine biological replicates. Error bars represent standard error of the mean.

After 72 h stimulation of Cal 27 cells with LPS + LTA, treatment with ML218 HCl and capsaicin resulted in 41.7% ± 3.1% and 32.2% ± 1.1% apoptotic cells, respectively. However, the number of apoptotic cells was reduced to 6.6% ± 0.95% and 2.98% ± 0.3%, respectively, if the additional bacterial antigens LPS + LTA were added simultaneously with the anti-cancer drugs. Treatment with ML218 HCl and capsaicin in the absence of bacterial antigens resulted in significantly more apoptotic cells than in the presence of bacterial antigens (*p* ≤ 0.001) (Figure 5).

### 2.5. Effect of ML218 HCl and Capsaicin Treatment on Gene and Protein Expression of Oral Cancer Cell Proliferation Factors

The *SOCS3* gene expressions were significantly higher in the capsaicin treatment group compared to ML218 HCl (*p* ≤ 0.001) (Supplementary Materials Figure S1). There was no significant *EGFR*, *STAT3* and *PI13KCA* gene expression difference among the treatment groups. A Western blot confirmed the results for PIK3 in protein expression. After 72 h, stimulation of Cal 27 cells with LPS + LTA, ML218 HCl treatment showed indications of higher protein expressions of EGFR and STAT3, and loss of the protein expression of SOCS3 (Supplementary Materials Figure S2). The capsaicin data reported in these figures are from our earlier study [3].

### 2.6. The Effect of ML218 HCl and Bacterial Antigens on Cal 27 TNFα Production

**Figure 6:**
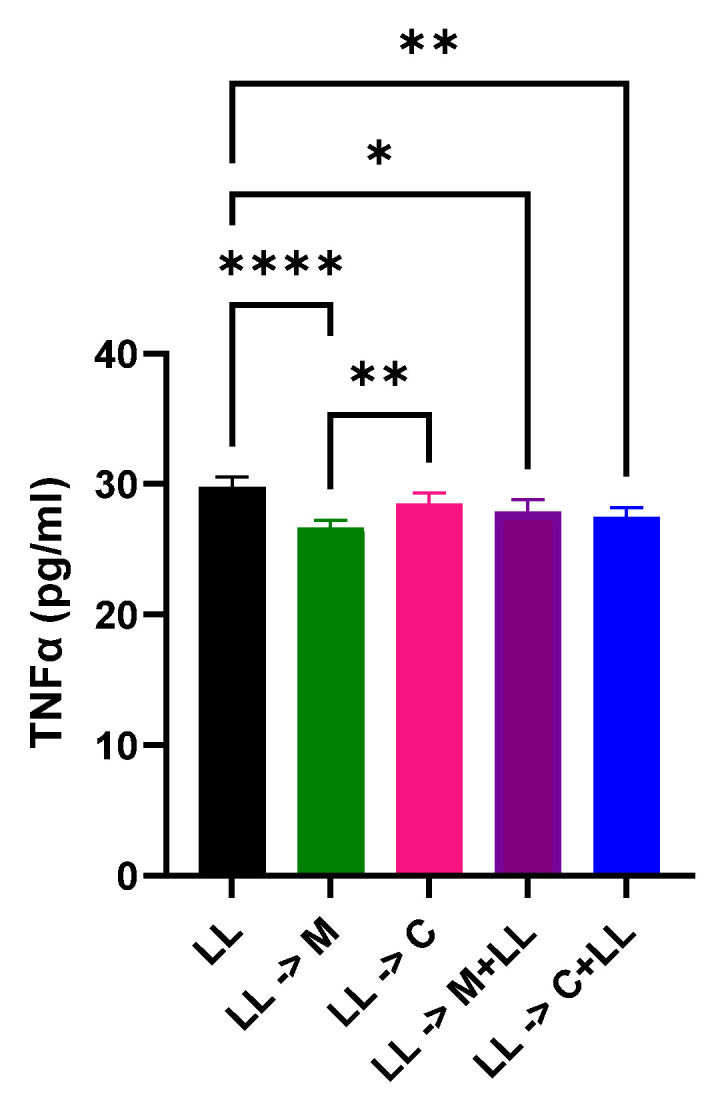
TNFα ELISA. Comparison of TNFα (pg/mL) under different treatment conditions. After 72 h stimulation of Cal 27 cells with LPS + LTA, Cal 27 cells were treated with drugs for 24 h in the absence or presence of bacterial antigens. M: ML218 HCl, C: capsaicin [3], LL = LPS + LTA. Letter before the arrow represents pre-stimulation, e.g., LL-> M is LL pre-stimulation and then treatment with ML218 HCl. * *p* ≤ 0.05, ** *p* ≤ 0.01, **** *p* ≤ 0.0001 is statistically significant. *n* = nine biological replicates. Error bars represent standard error of the mean. All of the columns look rather the same although they are mathematically different.

Treatment with ML218 HCl reduced Cal 27 TNFα production significantly (*p* ≤ 0.0001) (Figure 6). Addition of ML218 HCl to the pre-stimulated cells showed lower TNFα production compared to capsaicin (*p* ≤ 0.01) (Figure 6). However, the effect of ML218 HCl on TNFα production was annulled by the presence of bacterial antigens, similar to the effect of capsaicin [3] (Figure 6).

## 3. Discussion

Changes in calcium signalling affect cancer cell proliferation [12] as the influx of calcium upregulates various proliferation pathways [5]. It has been shown that the voltage-gated calcium channel and the receptor-dependent calcium channel facilitated the entry of calcium into cancer cells and led to their proliferation [13]. There might also be crosstalk among calcium channel receptors, since TRPV1 activation has been shown to downregulate voltage-gated calcium channels [14].

Titration of ML218 HCl identified the maximum clinically relevant drug concentration that could be used without adversely affecting normal cells, which was 100 µM for ML218 HCl, compared to 150 µM for capsaicin [3]. In the absence of antigenic stimulation, these doses of ML218 HCl and capsaicin reduced metabolism of Cal 27 cells by 100% and killed 70 and 65% of Cal 27 oral cancer cells, respectively. The addition of bacterial antigens during cell culture decreased the efficacy of drug treatment; however, the effect varied with the timing of antigen addition. The addition of antigen to non-stimulated Cal27 cells at the time of treatment decreased metabolic inhibition and decreased cell death for ML218 HCl, as reported for capsaicin [3]. Prior stimulation of Cal27 cells with antigen preceding the addition of ML218 HCl had no effect on efficacy, while it decreased capsaicin efficacy [3]. However, the combination of longer pre-stimulation Cal27 cells (72 h) and the addition of antigens at the time of ML218 treatment reduced efficacy. Generally, the effect of combined antigens was greater than LPS alone. Taken together, these results suggest that the presence of the bacterial antigen lowered the inhibition percentage of calcium channel drugs, and combined LPS+LTA acted synergistically to lower the effect of calcium channel drugs on Cal 27. This is similar to the previous findings of other researchers [15], with ML218 HCl performing better than capsaicin.

Following 72 h of bacterial antigen stimulation and treatment with calcium channel drugs, in the presence or absence of antigenic stimulation, the relative fold gene expression of *PI3KCA*, *EGFR*, and *STAT3* did not differ significantly among the treatment groups or from control Cal 27 cells (Supplementary Materials Figure S1). Similarly, pre-antigen stimulation plus drug treatment in the presence of antigen produced no change in *SOCS3* expression, although *SOCS3* expression was significantly increased in Cal 27 cells treated with capsaicin in the absence of bacterial antigens, compared to a modest increase for ML218 HCl. This difference is not unexpected as gene expression depends on both the metabolic rate of cells and the number of cells tested, and ML218 HCl treatment resulted in 100% inhibition and more cell death than capsaicin treatment under these same conditions.

In the Western blot analysis, cells treated with ML218 HCl after bacterial antigen stimulation showed the highest STAT3 and EGFR protein expression among treatments, but there was no difference between SOCS3 expression in ML218 HCl and capsaicin-treated pre-stimulated cells (Supplementary Materials Figure S2). Previous research has reported that overexpression of STAT3 turns STAT3 from anti-apoptotic to pro-apoptotic [16]. The current findings suggest that EGFR and STAT3 may be engaged in crosstalk in Cal 27 cells. Similar observations have been reported previously, where hyperactivation of EGFR mediates apoptosis via STAT3 [17]. Furthermore, none of the treatments showed any significant difference in PI3K protein expression. This may corroborate the previous finding that EGFR hyperactivation may result in the lowering of cell viability, independent of the PI3K/Akt pathway [18].

Based on the percentages of caspase 3/7-positive cells, ML218 HCl showed higher apoptosis compared with capsaicin under different bacterial antigen treatment conditions. This suggests that the voltage-gated calcium channel blocker was more potent in restricting the proliferation of Cal 27 than the TRPV1 agonist.

Functional T-type calcium channels are required for tumour growth and proliferation [19]. Previously it was reported that the blockade of T-type calcium channels in glioblastoma resulted in restriction of the progression of glioblastoma [20]. The findings of the current study along with the literature support the notion that Calcium oscillations (perturbations in calcium signalling) result in apoptosis of cancer cells by reducing the influx of calcium [4] by ML218 HCl (T-type calcium channel blocker) and an overload of calcium [5] as a result of excessive influx of Calcium by the TRPV1 agonist capsaicin. However, to date no study has compared the apoptotic efficiency of both calcium channel drugs in the same cell line.

The lowering of tumour suppressor protein SOCS3 is also consistent with the apoptotic percentage findings in this study, as all the tumour suppressor proteins were directly or indirectly related to the upregulation of the intrinsic pathway proteases caspase 3 and caspase 7 [21]. Also, as previously mentioned, hyperactivation of EGFR and STAT3 has been associated with increased functioning of the apoptotic machinery, which also upregulates caspase 3/7 activity [14]. This is also consistent with the caspase 3/7 percentage findings for Cal 27 treated with ML218 HCl after LPS + LTA stimulation.

LPS activates TNFα, leading to inflammation-induced cancer cell proliferation [22]. It is also associated with the proliferation and progression of cancer cells [23]. It has been previously shown that TNFα cross-talks with other proliferation and tumour suppression proteins [24].

This study showed that cells treated with ML218 HCl after bacterial antigen stimulation had the lowest TNFα, which may be attributed to overexpression of STAT3 and the loss of function of SOCS3. This is similar to previous findings that abrogation of SOCS3 function results in hyperactivation of STAT3 in macrophages [14].

ML218 HCl proved to be much more efficient than capsaicin in restricting Cal 27 proliferation. It is possible that the abovementioned scenario was orchestrated by TNFα or STAT3/EGFR interaction. Therefore, in future, a co-immunoprecipitation experiment could be performed to identify protein–protein interactions involving these proteins. Finally, since bacterial antigens have mostly been seen to affect immune cells, future studies using an immune cell-cancer cell co-culture model, or a tissue study on T-cell phenotypic changes should be undertaken to elucidate the role of bacterial antigens and calcium channel drugs thoroughly. The cell viability study for Cal 27 was only carried out at the maximum tolerated concentration of ML218 HCl by normal OKF6 cells. A future study could extend this report to see the effect of different ML218 HCl concentrations on the cell viability of Cal 27.

The determination of ML218 HCl concentrations was done with the help of a RealTime MT Glo cell viability assay. The cells were treated with drugs for 24 h. The major limitations to this approach are that: (a) it is not known whether treating the cells with a lower concentration, or the same concentration for a shorter time, would have been more effective in producing more apoptotic cells. Other assays such as Annexin V flow cytometry could have been used to determine the duration and concentration of the drug to achieve the best outcome; (b) the RealTime MT Glo cell viability assay is primarily a metabolic assay. It has inherent limitations, including that it could not predict the percentage of live or dead cells after treatment with drugs and antigens.

The percentage cell death caused by ML218 HCl in the presence and absence of stimulants was mainly based on the Trypan blue assay. Since oral cancer cells are of the epithelial-like adherent type, trypsin was used to detach the cells. Two drawbacks of this procedure that might have affected the overall outcome are that: (a) trypsin alone can disrupt the cell membrane of attached cells, and (b) live/dead cell counting under different conditions may result in incomplete detachment of the attached cells. Caspase 3/7 was analysed in the project, but other apoptotic markers may be involved in the antigen-oral cancer cell drug interaction.

## 4. Materials and Methods

Details of all materials and reagents used in this study are listed in Supplementary Materials Table S1.

### 4.1. Cell Lines and Culture Conditions

Oral cancer cells Cal 27 (American Type Culture Collection CRL-2095, Manassas, VA, USA) were used for this study, as we previously showed that LPS and LTA stimulation resulted in greater proliferation in Cal 27 cells than in other oral cancer cell lines (SSC4, SSC9, and SS25) or normal oral cell lines (*p* ≤ 0.001) tested [2]. Cal 27 cells were cultured using Dulbecco’s Modified Eagle Medium (DMEM) supplemented with 10% fetal bovine serum and 1% penicillin/streptomycin, while OKF6 (normal oral cells) were cultured in keratinocyte serum-free medium (KSFM) plus growth factors, as previously described [2]. Cells were seeded at 5000 cells per well in a 96-well plate as previously described [2]. Each 75 cm^2^ flask of cells acted as a biological replicate, and each flask was obtained from three different cell passages. Experiments were repeated at different times. Throughout this report, *n* refers to the number of biological replicates.

### 4.2. ML 218 HCl and Capsaicin Concentration

Cal 27 cells were incubated with capsaicin (Sigma-Aldrich, St. Louis, MO, USA) and ML218 HCl (Tocris, Bristol, UK) at varying concentrations from 0 to 150 µM and 0 to 100 µM for 24 h, and cellular metabolism was determined by the MT Glo assay. We also checked the effect of ML218 HCl and capsaicin (reported earlier [3]) on normal oral cells, using the OKF6 cell line, to reconfirm that the chosen concentration of the drugs does not harm normal cells (that are realistically always present near cancer cells in vivo). In this report, we used our previously published capsaicin metabolism and viability data [3] for ease of comparison with the data reported here from ML218 HCl treatment condition.

### 4.3. Bacterial Antigens and ML218 HCl and Capsaicin Combination Tests

Bacterial antigens, LPS (from Escherichia coli O111:B4, Sigma Aldrich, St. Louis, MO, USA) and LTA (from Streptococcus pyogenes, Sigma Aldrich, St. Louis, MO, USA) were used at their previously found optimum concentration of 5 µg/mL [1], ML218 HCl at 100 µM and capsaicin at 150 µM. Cal 27 cells were plated in a 96 well flat-bottomed plate at 5000 cells/well and stimulated with bacterial antigens LPS/LPS + LTA for 0, 24 and 72 h prior to treatment with ML218 HCl or capsaicin in the presence or absence of additional antigenic stimulation for 24 h. Then, cellular metabolism and viability were determined. Cal 27 cells without ML218 HCl or capsaicin treatment were used as control cells for each condition.

### 4.4. Inhibitive Effect of ML218 HCl and Capsaicin Treatment on Oral Cancer Cell Metabolism

Cell metabolism was determined using the real-time Glo MT cell viability assay (Promega) according to the manufacturer’s instructions. The percentage of inhibition of metabolism of capsaicin and ML218 HCl treatment was calculated as:

% Inhibition = [(Luminescence of the control cells − Luminescence of the treated cells) ÷ Luminescence of the control cells] × 100

### 4.5. Cell Viability Assay

Cell viability was determined by the Trypan blue exclusion assay. Cells were detached by the addition of 40 µL of 0.25% trypsin EDTA solution (Sigma, St. Louis, MO, USA), mixed in an equal volume of 0.4% Trypan blue (Invitrogen, Waltham, MA, USA), and live and dead cells were counted.

### 4.6. Apoptotic Assay

Caspase 3/7-positive cells are a marker of apoptosis. Cell Event Caspase-3/7 Green Detection Reagent (Invitrogen, Waltham, MA, USA) was used for live cell imaging and to estimate the total number of apoptotic cells, and 5000 cells (Cal 27) per 96-well plate (transparent, flat-bottomed) were plated and subjected to different treatments. After the incubation period for each treatment, the medium was carefully removed, and cells were washed once with 1 × PBS, then 100 µL of 7.2 µM Cell Event Caspase-3/7 Green Detection Reagent in 1 × PBS + 5% FBS was added to each well of the 96-well plate and incubated at 37 °C/5% CO_2_ for 30 min. The cells were imaged using the FITC filter sets, as the maximum excitation/emission for the Cell Event Caspase-3/7 Green Detection Reagent is 502/530 nm. Caspase3/7 apoptotic analysis was carried out using ImageJ version 1.52a software (National Institutes of Health, Bethesda, MD, USA, http://imagej.nih.gov/ij (accessed on 15 October 2021)). The treatment groups used for the apoptotic assay, qPCR, Western Blot, and TNFα ELISA were Cal 27 cells stimulated with bacterial antigens LPS + LTA for 72 h prior to treatment with ML218 HCl or capsaicin in the presence or absence of additional antigenic stimulation for 24 h.

### 4.7. Reverse Transcription Quantitative PCR for Proliferation Factors

Reverse transcription quantitative PCR was conducted for proliferation factors *SOCS3*, *EGFR*, *STAT3*, *GAPDH* and *PI3KCA*. RNA extraction and RT-qPCR and analysis were performed as described previously [9]. Briefly, total RNA was extracted with the Invitrogen Trizol Plus RNA purification kit. SSIV VILO Master Mix W/EzDNase (Invitrogen, Waltham, MA, USA) was used to reverse transcribe the RNA and qPCR conducted using Power-up SYBR Master Mix (Invitrogen, Waltham, MA, USA). Primer pair sequences are listed in Supplementary Materials Table S2. The house-keeping gene GAPDH (internal control) and the untreated Cal 27 cells were used to normalise the gene expression fold change in the treatment groups using the comparative Ct (∆∆Ct) method.

### 4.8. Western Blot

Ten grams of total protein from each bacterial antigen treatment condition was used for Western blotting. Primary antibodies were obtained from R&D Systems and used at the following concentrations: 0.1 g/mL human/mouse SOCS3 antibody, 1 g/mL human PI 3-kinase p110 antibody, 0.1 g/mL STAT3 Mouse anti-human, mouse, rat, 1 g/mL human EGFR antibody, 1:1000 human GAPDH. Blots were probed with secondary antibodies specific to each primary antibody source (detailed in Supplementary Materials) from R&D Systems at the following concentrations: 1:1500 anti-mouse IgG HRP conjugate, 1:1500 anti-goat IgG HRP conjugate, 1:1500 anti-rabbit IgG HRP conjugate. High-resolution images were acquired using the Chemidoc MP Imaging system and processed using Image Lab version 6.0 (Bio-Rad Laboratories, Hercules, CA, USA). The band densities of the product of the housekeeping gene, GAPDH (internal control) and untreated Cal 27 cells, were used to normalise the protein expression in the treatments.

### 4.9. TNFα ELISA

A Human TNFα ELISA kit (Invitrogen) was used to determine TNFα levels under different treatment conditions according to the manufacturer’s instruction.

### 4.10. Statistical Analysis

All statistical analyses were performed using GraphPad Prism version 9 (GraphPad Software, San Diego, CA, USA). The data were tested for normality of distribution using the D’Agostino and Pearson omnibus normality test. One-way or two-way analysis of variance (ANOVA) Kruskal–Wallis test multiple comparisons on mean ranks were performed in the oral cell proliferation and suppression, metabolism, viability and gene expression analyses. The probability threshold of *p* < 0.05 was considered statistically significant.

## 5. Conclusions

Previously, ML218 has been used only in research related to neurodegenerative disorders. To the best of our knowledge, this is the first report on the use of this voltage-gated calcium channel drug, ML218 HCL, in oral cancer research. The successful reduction in cell viability and metabolism by ML218 HCl is a novel finding of this study. We encourage further research in the field of voltage-gated calcium channels in oral cancer research. This study further strengthens the bacterial antigen combination drug strategy and sets a paradigm for future development of novel pharmacological management of oral squamous cell carcinoma. Thus, this report can serve as a platform to redesign the pharmacological treatment strategy for oral squamous cell carcinoma (OSCC) to include the concomitant use of antibiotics/prophylactic measures, along with chemotherapeutic drugs.

## Data Availability

The data presented in this study are available in Supplementary data file.

## Supplementary Materials

The following are available online at https://www.mdpi.com/article/10.3390/ijms222212559/s1.
