# Supplementary material for: ML218 HCl Is More Efficient Than Capsaicin in Inhibiting Bacterial Antigen-Induced Cal 27 Oral Cancer Cell Proliferation
Source: Int J Mol Sci. 2021 Nov 22;22(22):12559. doi: 10.3390/ijms222212559 (PMC8625738; doi:10.3390/ijms222212559)
Supplement: Supplementary file 1 [file ijms-22-12559-s001.zip › ijms-1462323-supplementary.pdf]

ML218 HCl is more efficient than Capsaicin in Bacterial Antigen induced Cal 27 oral cancer cell proliferation

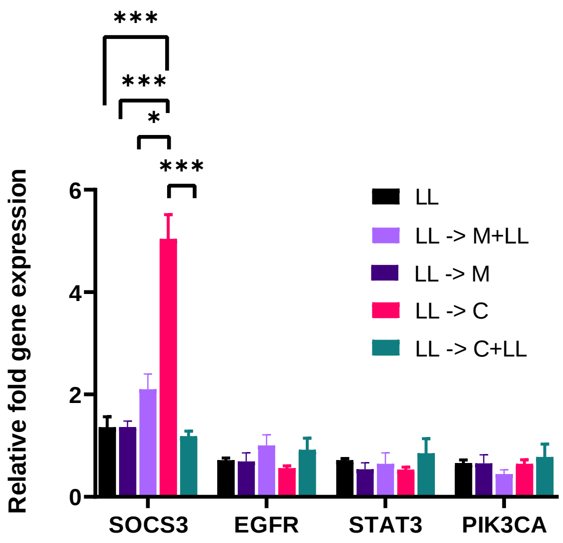

Figure S1. Expression of tumour suppressor and proliferation-related proteins by RT-qPCR analysis in Cal 27. All the gene expressions are relative to Cal 27 cells without bacterial antigen stimulation and ML2018 HCl or capsaicin treatment. M: ML218 HCl, C: capsaicin, LL = LPS + LTA. Letter before the arrow represents pre-stimulation, e.g., LL-> M is LL pre-stimulation and then treatment with ML218 HCl. * $p \leq 0.05$, *** $p \leq 0.001$ is statistically significant. $n = 9$ biological replicates. Error bars represent standard error of the mean.

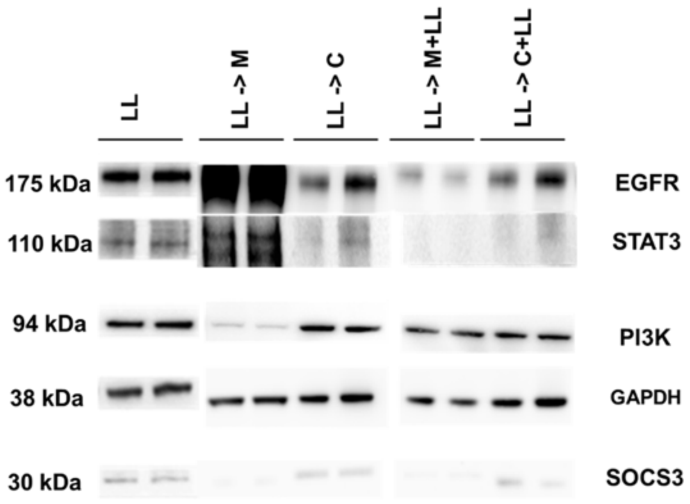

Figure S2. Western blot image of different tumour suppressor and proliferation related proteins in Cal 27. Cal 27 cells were stimulated with LPS + LTA (LL) for 72 h, and then treated with ML218 HCl or capsaicin for 24 h in the absence (LL -> M / LL -> C) or presence (LL -> M+LL / LL -> C+LL) of oral bacterial antigens. M = ML218 HCl, C = capsaicin, LL = LPS + LTA. Letter before arrow represents pre-stimulation e.g., LL -> M is LL pre-stimulation and then treatment with ML218 HCl.

Table S1: Detail of materials and reagents used in the study.

Reagent/kit	Manufacturer	LOT	Catalogue No.	Description
**Growth medium and buffers for the cell culture**				
Keratinocyte-Serum Free Medium (K-SFM)	Life Technologies	-	1700504	Contains 2.5 µg human recombinant epithelial growth factor (EGF) (0.035 µg/µl), catalogue No. 10450-013, LOT 1969988 and 25 mg bovine pituitary extract (15.1 mg/ml), catalogue No. 13028-014, LOT 2014313
Foetal bovine serum (FBS)	Life Technologies	-	10099141	Endotoxin level: ≤ 5 EU/ml.<br>Haemoglobin level: ≤ 30 mg/dL (levels routinely ≤ 25 mg/dL).
1X phosphate buffered saline (PBS)	Gibco	-	20012-027	1.5 mM KH_2PO_4; 155.2 mM Cl; 2.7 mM $Na_2HPO_4.7H_2O$ (pH 7.2)
Trypsin-EDTA	Sigma Life Sciences	T4049	SLBZ7364	0.25%, bioreagent 2.5 gm porcine trypsin and 0.2 gm EDTA; 4 Na per litre of Hank's balanced salt solution with phenol red
Dulbeccos Modified Eagles Medium (DMEM)	Gibco	-	11960-044	4,500 mg/L glucose, sodium pyruvate and sodium bicarbonate
Penicillin–streptomycin (P/S) solution	Life Technologies	2068825	15140-148	10,000 units/ml penicillin and 10,000 µg/ml streptomycin

Reagent/kit	Manufacturer	LOT	Catalogue No.	Description
Rosewell Park Memorial Institute (RPMI) 1640 medium	Gibco	1394048	21879-076	Without L-glutamine, and HEPES, with phenol red. Contains biotin, vitamin B12, PABA, vitamin inositol and choline. Used sodium bicarbonate buffer (2.0 g/L), and therefore required 5–10% CO_2 environment to maintain physiological pH
**Freezing of the cells**				
Tenak Slim tube cryogenic vials	Fisher Scientific	290992017	TE78315	RNAse–DNAse-free, 2-ml cryogenic vials
Dimethyl sulfoxide (DMSO)	Sigma Life Sciences	RNBH8443	D2650	Organic solvent, $(CH_3)_2SO$
**Drugs and bacterial antigens**				
LPS 25 mg	Sigma-Aldrich	-	L2630	Lipopolysaccharides from *Escherichia coli* O111:B4, water soluble
PHA 20 mg	Sigma-Aldrich	37614100	11082132001	Phytohemagglutinin-M (PHA-M) from *Phaseolus vulgaris*, water soluble
LTA 5 mg	Sigma-Aldrich	049M4155V	L3140	Lipoteichoic acid from *Streptococcus pyogenes*, water soluble
Capsazepine 5 mg	Sigma-Aldrich	039K4615	C191	N-[2-(4-Chlorophenyl) ethyl]-1,3,4,5-tetrahydro-7,8-dihydroxy-2H-2-benzazepine-2-carbothioamide, polar compound soluble in organic solvent

Reagent/kit	Manufacturer	LOT	Catalogue No.	Description
ML218 HCl (hydrochloride) 10 mg	Tocris	1A/226248	RDS450710	3,5-Dichloro-N-[[(1α,5α,6-exo,6α)-3-(3,3-dimethylbutyl)-3-azabicyclo [3.1.0] hex-6-yl] methyl]-benzamide hydrochloride, polar compound soluble in organic solvent
Capsaicin 250 mg	Sigma-Aldrich	MKBV4243V	360376	8-Methyl-N-vanillyl-trans-6-nonenamide, polar compound soluble in organic solvent
CID16020046 5 mg	Sigma-Aldrich	093M4730V	SML0805	4-[4,6-Dihydro-4-(3-hydroxyphenyl)-3-(4-methylphenyl)-6-oxopyrrolo[3,4-c] pyrazol-5(1H)-yl]-benzoic acid, polar compound soluble in organic solvent
Calcium chloride	Univar	D3247	127/500 g	$CaCl_2.2H_2O$ supplied as white powder. Water soluble; pH 5% solution at 25°C–4.5–8.5
**Proliferation assay**				
RealTime-Glo MT Cell Viability Assay	Promega	0000383765	G9712	10 ×100 reactions
MT Cell Viability Substrate, 1000X	Promega	0000362418	G971A	10 µl solution supplied in opaque tubes
NanoLuc Enzyme, 1000X	Promega	0000362418	E499A	0.4 mg/ml
Trypan blue stain 0.4%	Invitrogen	2117644	T10282	Trypan blue 0.4% to identify dead cells via colorimetric detection; supplied as 21 ml vials
Crystal violet acetate	Sigma Aldrich	69H3634	C5042	9(amino-5-imino-5H-benzo[a] phenoxyazine acetate salt suppled as powder

Reagent/kit	Manufacturer	LOT	Catalogue No.	Description
Methanol	Chem-Supply	UN1230	MA004-2.5L-P	Analytical reagent
Cell Countess cell-counting chamber slides	Invitrogen	I31A9 Q422	C10283	For live cell and dead cell percentage via calorimetric detection
**Protein estimation**				
Pierce BCA Protein Assay kit	Thermo Fisher Scientific	UD2969	23225	Two-component, high-precision, detergent-compatible protein assay for determination of protein concentration
**Kinase profiling**				
Proteome Profiler Human Phospho-Kinase Array kit	R&D Systems	P236166	ARY003C	8 arrays (4 Part A, and 4 Part B), 1 transparency overlay, 1 8-well multi-dish and reagents
Human Phosphokinase array	R&D Systems	1569148	899190	8 nitrocellulose membranes (4 Part A, 4 Part B) each containing antibodies printed in duplicate
Array Buffer 1	R&D Systems	P173293	895477	21 ml of buffered protein base with preservatives
Array Buffer 2 5X Concentrate	R&D Systems	P200594	895478	21 ml of concentrated buffered protein base with preservatives
Array Buffer 3	R&D Systems	P184800	895008	21 ml of buffered protein base with preservatives
Wash Buffer Concentrate	R&D Systems	P230760	895003	2 vials (21 ml/vial) of a 25-fold concentrated solution of buffered surfactant with preservative
Detection Antibody Cocktail A	R&D Systems	1568310	899188	1 vial of biotinylated antibody cocktail; lyophilised

Reagent/kit	Manufacturer	LOT	Catalogue No.	Description
Detection Antibody Cocktail B	R&D Systems	1568313	899189	1 vial of biotinylated antibody cocktail; lyophilised
Streptavidin-HRP	R&D Systems	1549191	893019	200 µl of streptavidin conjugated to horseradish peroxidase (HRP)
Chemi Reagent 1	R&D Systems	P222081	894287	2.5 ml of stabilised hydrogen peroxide with preservative
Chemi Reagent 2	R&D Systems	P222083	894288	2.5 ml of stabilised luminol with preservative
8-well multi-dish	R&D Systems	-	607591	Clear 8-well rectangular multi-dish
Transparency overlay template	R&D Systems	-	608281	1 transparency overlay template for coordinate reference
**Determination of proliferation related gene expression**				
Pure Link RNA mini kit	Invitrogen	2137737	12183018A	Total RNA extraction
Trizol reagent	Invitrogen	260702	15596026	Total RNA extraction
Ethyl alcohol	Sigma Aldrich	SHBH7551	E7023-1L	CH_3CH_2OH; pure
SSIV VILO Master mix W/ EzDNASE	Invitrogen	00831746	11766050	DNA digestion and Reverse transcription PCR
POWRUP SYBR Master Mix, 5 ml	Invitrogen	00799448	A25742	Real-time PCR Master Mix
Chloroform	BDH AnalaR	19073	VWRC22711.260	$CHCl_3$, contains 1% v/v of ethanol as preservative
MicroAmp Fast plate	Applied Biosciences, Life Biosystems	-	4346907	96-well reaction plate (0.1 ml)
Optical adhesive cover	Applied Biosystems, Life Technologies	-	4360954	qPCR-compatible optical adhesive covers
**Western blot**				

Reagent/kit	Manufacturer	LOT	Catalogue No.	Description
Human/Mouse SOCS-3 MAb (Clone 516919), 25 µg	R&D Systems	CCDL0219051	MAB5696	Purified mouse monoclonal IgG
Human/Mouse/Rat STAT3 MAb (Clone 232209), 25 µg	R&D Systems	JXW041912A	MAB1799	Purified mouse monoclonal IgG
Human PI 3-Kinase p110 beta MAb (Clone 269020), 25 µg	R&D Systems	VCK042001A	MAB2686	Purified mouse monoclonal IgG
Anti-G3PDH/GAPDH (T0893), 20 µL	R&D Systems	20481	2275-PC-020	Polyclonal rabbit antibody
Human EGF R/ErbB1 Polyclonal Ab, 25 µg	R&D Systems	AUC1118111	AF231	Affinity purified goat IgG
Novex Sharp pre-stained protein standard	Life Technologies	2115579	LC5800	consists of 12 pre-stained protein bands in molecular weight range 3.5–260 kDa
Clarity Western ECL Substrate	Bio-Rad	102031366<br>102031363	1705060	Supplied in two parts: peroxide solution and luminol/enhancer solution
Ponceau S solution	Sigma-Aldrich	SLCB3855	P7170-1L	Bioreagent 0.1% (w/v) supplied in 5% acetic acid: $C_{22}H_{12}N_4Na_4O_{13}S_4$
Anti-goat IgG HRP conjugate	R&D Systems	XGD10161011	HAF009	Secondary antibody specific to primary antibody source
Anti-rabbit IgG HRP conjugate	R&D Systems	FIN1819021	HAF008	Secondary antibody specific to primary antibody source
Anti-mouse IgG HRP conjugate	R&D Systems	WVA00919011	HAF018	Secondary antibody specific to primary antibody source

Reagent/kit	Manufacturer	LOT	Catalogue No.	Description
NuPAGE LDS sample buffer (4X)	Invitrogen	1981103	NP0007	Used to prepare protein samples for denaturing gel electrophoresis with Bis-Tris or Tris-Acetate gels. It contains lithium dodecyl sulfate, pH 8.4; contains Coomassie G250 and Phenol Red
NuPAGE 10% bis-tris gel	Invitrogen	19050170	NP0302BOX	1 mm × 12 well
NuPAGE 10% bis-tris gel	Invitrogen	19071070	NP0301BOX	1 mm × 10 well
20X NuPAGE MOPS SDS Running Buffer	Invitrogen	-	NP0001	500 ml contains 50 mM MOPS, 50 mM Tris Base, 0.1% SDS, 1 mM EDTA, pH 7.7
**Apoptotic assay**				
CellEvent Caspase-3/7 Green Detection Reagent 25 µl	Invitrogen	2119122	C10723	2.0 mM solution in DMSO
**TNF α enzyme-linked immunosorbent assay (ELISA)**				
Human TNF α ELISA kit	Invitrogen	225688-001	KHC3011	Supplied with all the reagents

Table S2: Primers used in RT-qPCR for proliferation factors.

Gene Name	Accession Number	Sequence (5'-3')	Position	Exon – Exon	PCR product length (bp)
SOCS3	NM_003955.4	GCGCGAAGGCTCCTTTGTG	191		135
		GGGGGGCTGGTCCCGAATC	336	330/331	
STAT3	NM_139276.2	GGACATCAGCGGTAAGACCC	2118	2128/2129	168
		CTCTGGCCGACAATACTTTC	2310		
EGFR	NM_005228.5	AGCTACGGGGTGACTGTTTG	2956	2962/2963	106
		GAACTTTGGGCGACTATCTG	3114		
GAPDH	NM_002046.7	GACAGTCAGCCGCATCTTCT	21		181
		ACCAAATCCGTTGACTCCGA	112	105/106	
PI3KCA	NM_006214.4	TGG GGATGATTTACGGCAAG	2732	2739/2740	129
		TCCCACACAGTCACCGATTGA	2861		
